# Optimizing the predictive power of depression screenings using machine learning

**DOI:** 10.1177/20552076231194939

**Published:** 2023-08-29

**Authors:** Yannik Terhorst, Lasse B Sander, David D Ebert, Harald Baumeister

**Affiliations:** 1 Department of Clinical Psychology and Psychotherapy, Institute of Psychology and Education, 9189 University Ulm, Ulm, Germany; 2 Medical Psychology and Medical Sociology, Faculty of Medicine, 385631 University of Freiburg, Freiburg, Germany; 3 Department for Sport and Health Sciences, Chair for Psychology & Digital Mental Health Care, 520956 Technical University of Munich, Munich, Germany

**Keywords:** Major depressive disorder, diagnosis, machine learning, digital health, health care

## Abstract

**Objective:**

Mental health self-report and clinician-rating scales with diagnoses defined by sum-score cut-offs are often used for depression screening. This study investigates whether machine learning (ML) can detect major depressive episodes (MDE) based on screening scales with higher accuracy than best-practice clinical sum-score approaches.

**Methods:**

Primary data was obtained from two RCTs on the treatment of depression. Ground truth were DSM 5 MDE diagnoses based on structured clinical interviews (SCID) and PHQ-9 self-report, clinician-rated QIDS-16, and HAM-D-17 were predictors. ML models were trained using 10-fold cross-validation. Performance was compared against best-practice sum-score cut-offs. Primary outcome was the Area Under the Curve (AUC) of the Receiver Operating Characteristic curve. DeLong's test with bootstrapping was used to test for differences in AUC. Secondary outcomes were balanced accuracy, precision, recall, F1-score, and number needed to diagnose (NND).

**Results:**

A total of k = 1030 diagnoses (no diagnosis: k = 775; MDE: k = 255) were included. ML models achieved an AUC_QIDS-16_ = 0.94, AUC_HAM-D-17_ = 0.88, and AUC_PHQ-9_ = 0.83 in the testing set. ML AUC was significantly higher than sum-score cut-offs for QIDS-16 and PHQ-9 (*ps* ≤ 0.01; HAM_D-17: *p* = 0.847). Applying optimal prediction thresholds, QIDS-16 classifier achieved clinically relevant improvements (Δbalanced accuracy = 8%, ΔF1-score = 14%, ΔNND = 21%). Differences for PHQ_9 and HAM-D-17 were marginal.

**Conclusions:**

ML augmented depression screenings could potentially make a major contribution to improving MDE diagnosis depending on questionnaire (e.g., QIDS-16). Confirmatory studies are needed before ML enhanced screening can be implemented into routine care practice.

## Introduction

In mental health care, an accurate, psychometrically sound assessment, and diagnosis in a timely manner is the key to treatment and optimized health care pathways.^1–5^ To achieve this goal, different assessment and diagnosis approaches exist ranging from structured clinical interviews (e.g. SCID)^6^ to self-report instruments (e.g. nine-item Patient Health Questionnaire (PHQ-9))^7,8^ and clinician rating scales (e.g. 16-item version of the Quick Inventory of Depressive Symptomology (QIDS-16)).^4,9^ Given the economic pressure of many health care systems,^10–12^ time-consuming diagnostic assessments like SCID seem difficult which may contribute to the issue of under-recognition.^13–16^ A feasible time-saving alternative to SCID is the use of self-report or clinician-rated screening instruments.^1,15,16^

For such screening instruments, traditionally, a cut-off value (e.g. for the sum scores of all items of the instrument) is determined by balancing the sensitivity and specificity (e.g. optimal Youden Index in a Receiver Operating Characteristic (ROC) curve).^7,17,18^ An individual participant data analysis with *N* = 44,318 participants demonstrated a sensitivity of 0.86 (95%-CI: 0.80 to 0.90) and specificity of 0.85 (95%-CI: 0.82 to 0.87) for such cut-off scores regarding the prediction of major depression with the PHQ-9.^19^ However, while these characteristics are impressive, it has also been shown that cut-off approaches compared to SCID can lead to biased diagnosis, highlighting the need for further optimization.^20^

In recent years, machine learning (ML) has been applied in many areas accompanied by a paradigm shift away from a priori human-defined solutions for problems like prediction tasks towards algorithmic solutions by ML methods automatically determining parameters to reach an optimal solution.^21,22^ In short, such ML algorithms try to find an optimal function to map the observed data and features to an output, while bias (=prediction error) and variance (=performance difference between known data—training data, and unknown data—testing data) are balanced.^22–24^ To achieve this, various ML models (e.g. random forest, neural nets) and their hyperparameters (e.g. learning rate) are usually tested and tuned. Doing so, ML has been shown to achieve high accuracy in many prediction tasks^21,22^ and may also offer a way towards optimized diagnostic procedures.

Given its high prevalence^2,25^ and impact on global health,^26–29^ ML-based diagnosis has been studied in particular for depression. However, while studies based neuroimaging,^30^ microRNA,^31^ RNA,^32^ peripheral blood markers,^33^ and other biomarkers^34^ show promising results in some cases, the necessary data for such biomarkers is often obtained with resource and cost-expensive methods. Hence, the development of ML prediction models based on data, which is easily and cost-efficiently obtainable, may offer a more promising way to improve diagnosis. As such, frequently available screening instruments like self-report questionnaires or clinician-rated instruments could be used in ML classifiers. However, currently, it is unclear to which extent their performance can be optimized by ML compared to existing best practice clinical cut-offs.

Therefore, the present study evaluates the potential of ML classifiers to detect a diagnosis of a major depressive episode (MDE) based on the items of commonly used screening instruments and evaluates their performance compared to established best-practice clinical sum-score approaches in which a diagnosis is given if a score threshold is exceeded. Given the impact of the chosen threshold on the accuracy (e.g. lower thresholds increase sensitivity and decrease specificity), both performance metrics across the spectrum of all possible thresholds and performance metrics for a single best-practice threshold (i.e. optimal balance between sensitivity and specificity) need be addressed. Accordingly, the following research questions will be answered:

1. Primary research question: Is there a significant difference between the Area Under the Curve (AUC) of the ROC across all possible class prediction thresholds in ML models and sum-score thresholds of the questionnaires?
2. Secondary research question: Is there a difference in the balanced accuracy, precision, recall, F1-score, and number needed to diagnose (NND) between ML models and sum-score approaches, if, respectively, the best threshold is applied (e.g. best class probability threshold compared to best-practice sum-score cut-off)?

## Methods

### Study design and sample

The present study is a secondary analysis of the large-scale pragmatic, observer-blinded randomized controlled trials WARD-BP (*N* = 210) and PROD-BP (*N* = 295).^35–38^ In short, the trials investigated the effectiveness of a digital intervention for depression in individuals with clinical or sub-clinical depressive symptoms, respectively. Both trials followed a parallel design with multiple assessment points providing self-reported, clinician-rated depression assessments and clinical diagnosis of depression (cf. Measures and Outcomes). Recruitment was conducted on-site in clinics and via letters in both RCTs. The primary data consisting of the diagnosis and corresponding questionnaires’ data were obtained for the present analysis. All MDE cases and no depression (=absence of any depressive disorder) cases were included.

All procedures have been approved by the ethics committee of the Albert-Ludwigs-University of Freiburg, Germany, and the data security committee of the German Pension Insurance. All participants provided written informed consent for participation in the studies. The studies were registered in the German Register for Clinical Trials (DRKS: DRKS00009272, registered on 14 September 2015; DRKS00007960, registered on 12 August 2015). Further details on the studies are provided in their study protocols and main result publications.^35–38^ The present study followed the STARD^39^ and TRIPOD^40^ reporting guidelines (see checklists in supplement 1).

### Measures and outcomes

**Ground Truth:** For the diagnosis of an MDE, the SCID for DSM-5 and SCID-5-RV module were used and conducted by trained and supervised psychologists. The training included at least four training interviews supervised by a licensed psychotherapist. The agreement between supervisors and trainees was excellent (κ > 0.90).^36,37^ Interviewers were all blinded toward the group allocation. Participants were not interviewed by the same clinical psychologist twice. Following this diagnostic procedure, cases with either an MDE (ground truth class = 1) or not any depressive disorder (ground truth class = 0) were used in the present analysis. Cases with depressive diagnoses other than MDE (i.e. persistent depressive disorder) were excluded.

**Self-report instruments:** The nine-item version of the PHQ-9 is a reliable, valid, and widely used screening instrument for depression with similar psychometric quality in its computerized version, which has been used in the included studies.^7,41^

**Clinician-rated instruments:** Besides the structured interview, the clinical psychologists rated the 17-item version of the Hamilton Rating Scale for Depression (HAM-D-17) and the QIDS-16.^9,42^ Mixed and good psychometric properties are reported for the instruments, respectively.^9,42–45^

### Analysis

We trained a range of candidate models to find the best mapping from the included features (e.g. questionnaire items) to the binary outcome of MDE status. Candidate models varied in: (a) model specifications (e.g. random forest, logistic regression); (b) feature sets (e.g. original items, extended features, demographic variables); (c) dimension reduction steps (e.g. principal component analysis); and (d) data imbalance handling (e.g. down sampling). Please see below for a detailed description. A stepwise combination from (a)–(d) resulted in a total of 261 candidate models for each questionnaire. The preprocessing and analysis code are published in the open science framework (https://osf.io/3hnvz/).

**General procedure:** For the training and testing split a random 75%/25% split was chosen. The training was conducted using 10-fold cross-validation with five repeats. Stratification of MDE status was used in data splitting and cross-validation. Proportions of the MDE status in the training and testing set can be found in supplement 2. The performance summary of all trained models is reported in supplement 3. The best fitting candidate model based on the highest ROC AUC from training was applied to the testing set for each respective questionnaire for primary and secondary outcomes (see below).

**Model specifications:** The following ML models were chosen as candidates: logistic regression (lr), extreme gradient boosted regression trees (XGB), single layer neural network (mlp), Naïve Bayes (nb), Bayesian additive regression trees (bart), multivariate adaptive regression splines (MARS), bagged MARS (bag_MARS), decision tree, bagged decision tree (bag_tree), linear (svm_l), radial basis (svm_r), and polynomial (svm_p) support vector machine, random forest (rf), and K-Nearest Neighbor (knn).^24,46^ Hyperparameters of the ML models were optimized by grid-search combined with an ANOVA racing method.^47^ In short, performance metrics (ROC AUC and J-Index) were determined for a set of tuning parameters for each of the models (25 candidate values for each hyperparameter). The racing method eliminated tuning parameter combinations that were unlikely to improve the model after an initial number of resamples using a repeated measure ANOVA model. For more details on the applied grid search racing approach see.^47^ A list of all tuned hyperparameters see supplement 4.

**Feature sets:** All candidate models were provided the item answers of each scale. In addition, candidate models were trained with an extended feature list (mean, median, variance, max, min, kurtosis, skewness, and frequency of each response option were created as features for each case), and demographic variables (age, gender).

**Dimension reduction:** Given the correlation between features, candidate models were trained without dimensionality reduction, supervised principal component analysis with partial least squares, and uniform manifold approximation and projection.^46^

**Data imbalance:** MDE status was unbalanced in the data set (see results). While this mirrors the “in the wild” prevalence of MDE with a majority of healthy individuals and a minority of individuals with MDE,^25^ imbalance can potentially affect the performance of ML.^48^ Hence, candidate models were trained without imbalance handling, under sampling,^46^ and Random Over-Sampling Example (ROSE) method.^49^ Under sampling and ROSE was only used in the training, but not in the testing subset to evaluate the “in the wild” performance of the trained classifiers in the testing set.

**Feature preprocessing:** We followed preprocessing recommendations by Kuhn and Silge^46^ and removed zero variance features, normalized features, applied orthogonal polynomial basis functions, and included interactions of features depending on the algorithms.

**Primary outcome:** The ROC and the corresponding AUC was the primary outcome. An ROC compares the true positive rate against the false positive rate at different thresholds of an outcome (e.g. the varying sum score cut-offs). The AUC of the ROC gives an aggregated measure of the performance across all possible thresholds of an outcome. An AUC of 0 would indicate that 0% of the predictions are correct, while an AUC of 1 would indicate 100% correct predictions.

The ROC AUC of the best ML models after training was compared in the testing set against the AUC of varying sum scores for each of the PHQ-9, HAM-D-17, and QIDS-16 instruments according to their respective scoring procedures.^7,9,42^ DeLong's test for two correlated ROC with *n* = 2000 bootstraps was used to test (two-sided) for a significant difference between the ML models and the traditional sum score cut-off approach.^50^ The type I error level for the comparison was set to 5%. Additionally, bootstrapped 95% confidence intervals (CI) for the true difference between the ROCs were calculated.

**Secondary outcome:** The ROC AUC provides a comprehensive performance estimate over the whole spectrum of possible prediction thresholds (e.g. best-practice clinical cut-offs for questionnaire sum scores, or predicted class probability in ML) and was hence chosen as the primary outcome. In contrast, the balanced accuracy, recall, precision, F1-score, and the NND (=the number of patients who need to be examined to correctly detect one person with depression) provide a performance metric for a single threshold. We calculated the balanced accuracy, recall, precision, F1-score, and NND in the testing set for the secondary outcome comparison based on the following rationale:

Firstly, all three questionnaire have established clinical cut-offs.^51,52^ However, there is an ongoing debate on the best sum score cut-offs for the instruments.^17,53,54^ For the present secondary analysis, we have chosen the following cut-offs PHQ-9 ≥ 10,^7,19^ HAM-D-17 ≥ 8,^54^ and QIDS-16 ≥ 6.^9^

Secondly, since the optimal cut-offs for PHQ-9, HAM-D-17 and QIDS-16 might be different in the present sample, we additionally determined the secondary outcome metrics for best-sample-specific cut-offs for the questionnaires in the training sample. Cut-offs were determined using the Youden Index.^18,55^

Thirdly, we calculated the optimal ML prediction threshold for the best ML candidate in the training set using Youden Index^18,55^ and calculated the secondary outcomes in the testing set based on this threshold.

### Software

All analyses were conducted in R.^56^ The tidymodels framework was used for ML training and analysis.^46^ For detailed session information for all R packages and versions see online supplement 5. Analysis code is available at: https://osf.io/3hnvz/.

## Results

**Figure 1.**
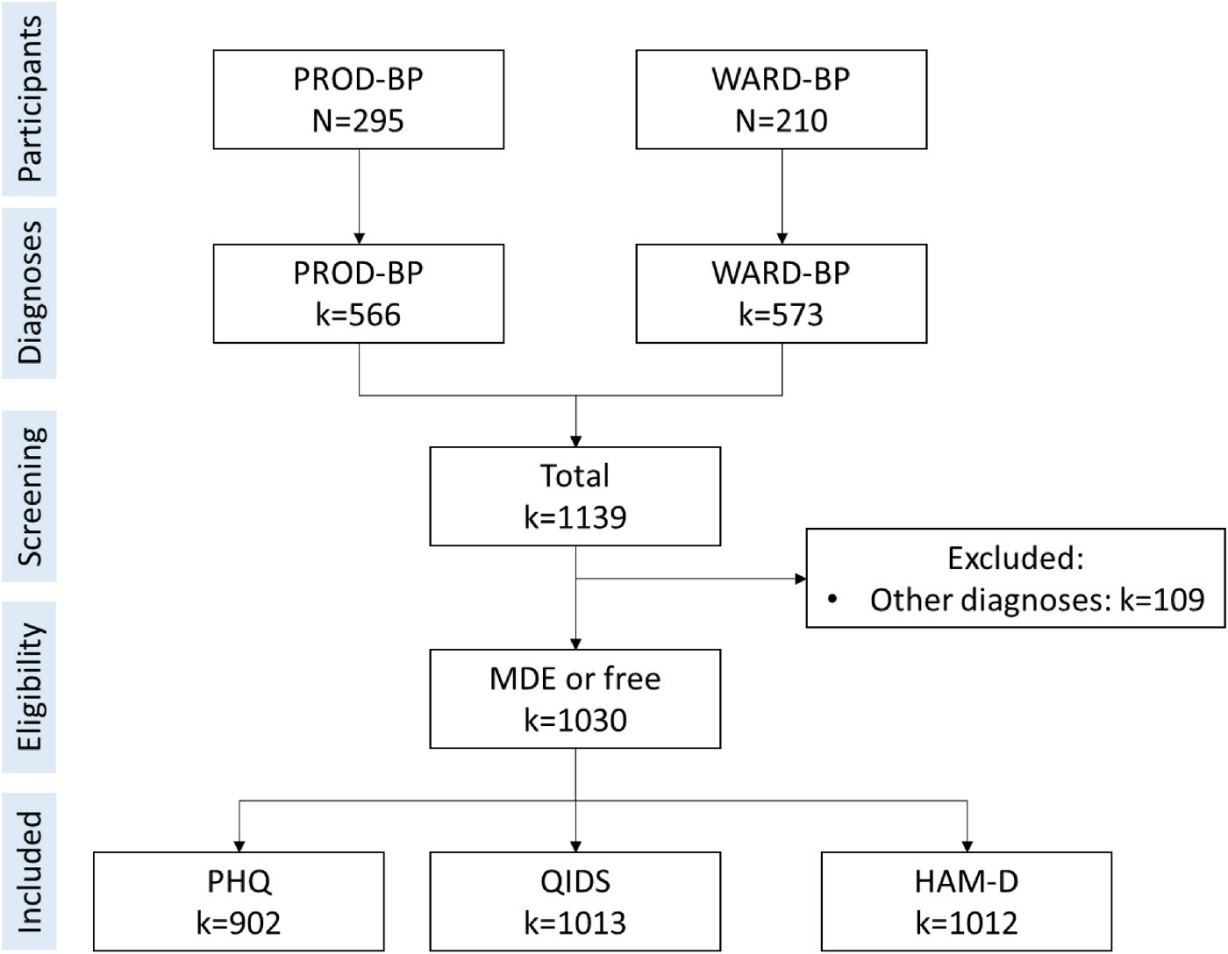
Included cases from WARD-BP and PROD-BP. *Note.* Questionnaire assessments (PHQ-9, QIDS-16, HAM-D-17) and diagnoses (SCID-V) were conducted at multiple time points over the course of the studies. Each assessment point was independent (i.e. clinical assessor was changed after each assessment and clinicians were blinded towards treatment condition and previous results).

The total number of *k* = 1030 diagnoses (non-depressed: 775; depressed: 255) were included (Figure 1). The gender distribution of the diagnoses was 629 female (61.1%) and 401 male (38.9%) diagnoses. The mean age was *M* = 51.80 (*SD* = 8.64, *min* = 22, *max* = 79). Depression severity across all diagnoses was *M* = 8.62 (*SD* = 4.65) based on self-reported PHQ-9 and based on clinician-ratings *M*_HAM-D_ = 8.72 (*SD*_HAM-D-17_ = 5.97) and *M*_QIDS-16_ = 6.57 (*SD*_QIDS-16_ = 4.44).

### Primary outcome

#### Clinician-rated instruments

The trained QIDS-16 classifier with only the QIDS-16 items as predictors yielded an AUC = 0.935 in the testing set. In comparison, the AUC using the traditional QIDS-16 sum score achieved an AUC = 0.901. DeLong's test showed a significant difference between the AUC favoring the ML approach (ΔAUC = 0.04, 95%-CI: 0.02 to 0.05, *p* < .001). A summary of feature importance in the prediction can be found in supplement 5. Adding the extended feature set, age, and gender to the model improved the AUC marginal, but without clinical relevance (AUC = 0.937, ΔAUC = 0.002 compared to ML with only QIDS-16 items).

For the HAM-D-17, a random forest model with the original HAM-D-17 items achieved the best performance of all candidate models. While the ROC AUC of the HAM-D-17 ML model was also higher (AUC = 0.876) in the testing set compared to the sum-score approach (AUC = 0.873), the difference was not significant (ΔAUC = 0.00, 95%-CI: −0.02 to 0.03, *p* = .847). A summary of the feature importance is displayed in supplement 5.

Self-report instrument:

For the PHQ-9, a Naïve Bayes model based on the PHQ-9 items and the extended features set significantly outperformed the traditional cut-off approach: AUC for the Naïve Bayes model was AUC = 0.83 compared to an AUC = 0.82 for sum scores (ΔAUC = 0.01, 95%-CI: 0.00 to 0.02, *p* = .009). Information regarding feature importance can be found in supplement 5.

See Table 1 for an overview of the primary outcome across all three questionnaires.

**Table 1.** Primary performance comparison AUC of ROC.

	AUC of ROC for the best ML	AUC of ROC for sum-score	ΔAUC	95%-CI of the difference	*p* ^a^
QIDS-16—basic^b^	0.935	0.900	0.035	0.02 to 0.05	<0.001
QIDS-16—extended^c^	0.937	0.900	0.036	0.02 to 0.06	<0.001
HAM-D-17	0.876	0.873	0.003	-0.02 to 0.03	0.847
PHQ-9	0.832	0.818	0.014	0.00 to 0.02	0.009

^a^ Two-sided bootstrap test for ROC curves within in the testing set, indicating the probability of whether the true difference in AUC is different from 0.

^b^ Logistic regression model containing only the QIDS-16 items as features.

^c^ Logistic regression model containing QIDS-16 items, the extended feature set (e.g. mean of items), age, and gender.

### Secondary outcomes

For further comparison the balanced accuracy, precision, recall, F1-score, and the NND were determined for the performance in the testing set following the rationale of best clinical thresholds, best sample-specific thresholds (QIDS-16 ≥ 9, HAM-D ≥ 11, PHQ-9 ≥ 9), and best class probability thresholds in the training set (QIDS-16: 0.28, HAM-D-17: 0.32, PHQ-9: 0.01).

Overall, the balanced accuracy achieved by the ML classifiers outperformed established clinical cut-offs. While precision or recall as a stand-alone metric favored established cut-offs in some cases, the harmonic mean of precision and recall (=F1-score) and balanced accuracy always favored the ML classifiers. Additionally, the NND favored the ML classifiers. For a summary of all secondary outcomes, see Table 2. For the calculation of additional performance metrics, the confusion matrix can be found in supplement 6.

**Table 2.** Secondary performance metrics.

	Classifier	Balanced accuracy	Recall	Precision	F1-score	NND
QIDS-16	ML—basic^a^	0.87	0.86	0.71	0.77	1.35
	ML—extended^b^	0.86	0.83	0.72	0.77	1.39
	Clinical sum-score cut-off	0.79	0.95	0.47	0.63	1.71
	Sample specific cut-off	0.82	0.78	0.65	0.71	1.56
HAM-D-17	ML	0.81	0.75	0.65	0.70	1.63
	Clinical sum-score cut-off	0.75	0.89	0.43	0.58	2.02
	Sample specific cut-off	0.79	0.77	0.58	0.66	1.72
PHQ-9	ML	0.76	0.79	0.49	0.61	1.93
	Clinical sum-score cut-off	0.76	0.79	0.49	0.60	1.95
	Sample specific cut-off	0.72	0.81	0.43	0.56	2.26

*Note.* All secondary outcomes refer to the performance in the testing set. Optimized thresholds were determined in the testing set.

^a^ Logistic regression model containing only the QIDS-16 items as features.

^b^ Logistic regression model containing QIDS-16 items, the extended feature set (e.g. mean of items), age, and gender.

## Discussion

The present study evaluated the potential of ML to enhance the performance of clinical assessment tools to detect MDE based on clinician-rated (QIDS-16, HAM-D-17) and self-report (PHQ-9) questionnaire data obtained in a pragmatic health care setting. The present results demonstrated that ML models like penalized logistic regression or naïve Bayes models can significantly increase the AUC of the ROC curve for the clinician-rated QIDS-16 (*p* ≤ 0.001) and the self-report PHQ-9 (*p *= .009). However, while the difference for QIDS-16 and PHQ-9 were significant, especially the difference for the QIDS-16 indicates clinically relevant improvements (e.g. Δbalanced accuracy = 8%, ΔF1-score = 14%, ΔNND = 21%) if the best class probability threshold is compared against current best practice clinical sum-score cut-offs. Underlining this finding, clinically relevant improvements were also observed for the QIDS-16 classifier in comparison to the best sample-specific cut-off (e.g. Δbalanced accuracy = 5%, ΔF1-score = 6%, ΔNND = 13%). However, in contrast the differences in PHQ-9—although statistically significant—are below clinical relevance (ΔROC AUC = 1%, Δbalanced accuracy = 0%, ΔF1-score = 1%, ΔNND = 1%), and for the HAM-D-17 we found no statistically difference in the ROC AUC, while seeing differences favoring ML, if the best class probability threshold for the ML classifier is compared to the clinical sum-score cut-off (Δbalanced accuracy = 6%, ΔF1-score = 12%, ΔNND = 19%).

A key issue in the field limiting ML in achieving high accuracy and potentially explaining the differences in the findings across the here evaluated questionnaires, is the noise and measurement error of the underlying questionnaires and items (e.g. caused by social desirability bias, recall bias, systematic and random measurement error bias, or confirmation bias).^57–59^ In particular, patient reported outcome measures are prone to some biases (e.g. social desirability) compared to instruments rated by trained clinicians (e.g. QIDS-16), which may contribute to the difference of improvement by ML based on the QIDS-16 and PHQ-9. However, eliminating some sources of bias by a clinician rating does not guarantee a reliable assessment. For instance, the reliability of the HAM-D-17 has been shown to be questionable, in particular for some items.^44,60,61^ Contributing to this, the HAM-D-17 is characterized by less precise wording regarding the frequency and duration of symptoms when compared against other instruments like the PHQ-9 or QIDS-16 (e.g. HAM-D-17 mood refers to if feeling states of sadness are reported without categorization of frequency or duration, compared to PHQ-9 and QIDS-16 specified responses whether sadness occurs “at all”, “several times”, “more than half the times”, “nearly all the time”).^9,42^ In addition, core symptoms of depression according to the DSM-5^6^ or ICD-11^62^ (i.e. hypersomnia or hyperphagia) are missing in the HAM-D-17 potentially further explaining an increase in noise and measurement error compared to PHQ-9 and QIDS-16.^4,9,42^ Applying more precise and highly reliable assessments in clinical practice like computer-adaptive tests may provide an opportunity to increase measurement quality of questionnaires and thereby potential of ML in future.^63–66^

Furthermore, the present study could be expanded by including other data sources like electronic health records, molecular biosignatures (e.g. epigenomics), environmental data (e.g. lifestyle), physiological data, and smart-sensing and digital phenotyping data providing unbiased objective behavioral data using sensors in our daily life from omnipresent smart devices (e.g. in smartphones, smart watches and other wearables), might be promising to further augment ML algorithms in their predictive accuracy and maximize the potential of complex data and ML.^34,67–75^ However, this goes hand in hand with higher effort and costs to collect such data. In particular, the QIDS-16 might be suited to provide ML-optimized MDE predictions even without such an extension. That said, for health care providers (e.g. physicians), who have access to various sources of health records, the combination of different sources in an ML augmented expert system assisting them in the diagnosis of mental (and other) disorders could be feasible and promising.^75^

In future, the application ML in the screening of depression and providing an instant prediction of the depression status based on the questionnaire data alone (e.g. based on QIDS-16)—or further augmented by objective sensing data, health records data, or biological parameters—may offer a way towards an accurate, time-efficient and thus feasible depression screening leading to earlier diagnosis and treatment initiation in resource-limited mental health care.^10,11,13,14^ If proven to be effective, this could have a major impact on highly prevalent mental disorders like depression and public health.

However, some limitations of the present study must be considered and targeted in future studies before the implementation of ML-optimized depression screening in clinical settings. Most importantly, the present study is an exploratory secondary analysis. We used 10-fold cross-validation in the present study.^76,77^ Accordingly, the algorithms were trained on a subset of the data (exploratory) and the final performance of the trained models was evaluated on previously unknown data (confirmatory). While this procedure mirrors the logic of a confirmatory study to some extent, it cannot replace the necessity of confirmatory experimental studies eliminating other potential sources of bias and effects. Clinical randomized controlled trials (e.g. comparing ML-enhanced routine care screening of MDE against screening as usual) are of utmost importance before it comes to ML-augmented screening tools or expert systems in routine care settings. Also, it must be ensured that any ML-enhanced medical application derived is fair and unbiased.^78–82^ While the exploration of age and gender as additional features in the present analysis provided no meaningful improvements and is speaking for the generalizability, further evidence is needed that the benefit of ML in screening accuracy and performance holds across different gender, ages, ethnic and cultural populations. Speaking of generalizability, the setting of the included RCTs also needs to be considered. While the diagnoses and data of the present study were obtained in a pragmatic healthcare setting and the exploration of ML-enhanced screening procedures based on real-world healthcare data is a strong suit of this study, the healthcare setting at hand is also very specific: All participants were recruited in orthopedic rehabilitation centers.^35–38^ The replication of the present findings in different settings is highly needed. Nonetheless, by highlighting the potential of ML-enhanced screening tools for some questionnaires widely used in clinical practice (i.e. QIDS-16), the present study makes an important first step toward clinical trials and lays the foundation for future clinical implementation. Building on this, future studies could also move away from a binary classification (e.g. depression-free vs. MDE) towards a multi-classification approach to also support the differential diagnosis (e.g. distinguishing between various mental disorders with similar symptoms). However, it also needs to be highlighted, that the current nosology in clinical practice only insufficiently reflects the heterogeneous nature of mental disorder.^83–85^ For instance, over 1000 unique symptom profiles with a frequency of 1.8% of the most common profile have been reported in 3703 outpatients.^84^ Furthermore, the poor reliability of diagnoses^86^ and the high comorbidity as a potential indicator of unitary disorders being split up into various diagnoses^83^ pose major limitations for research and practice. Investigating how ML can be applied in different systems (e.g. Hierarchical Taxonomy of Psychopathology^83^ or the Extended Evolutionary Meta-Model^87,88^) to predict clinical relevant symptomology and inform treatment processes would be a very valuable addition to this study.

## Conclusions

The present study evaluated the potential of ML to increase the performance of screening instruments for MDE. ML improved the performance of clinician-rated and self-report instruments (QIDS-16, PHQ-9). In particular, the optimization of the QIDS-16 indicated high clinical relevance. If proven to be effective in confirmatory studies, implementing ML-enhanced screening tools in clinical practice could significantly improve diagnostic procedures. Given that a timely and efficient diagnosis is key in healthcare, ML applications in mental health care may lay the foundation for optimized mental healthcare in future. However, further research how the potential of ML can be exploited (e.g. by including more data sources like socio-demographic, health records, biomarkers, and smart sensing data) and the implementation into routine care is highly needed.

## Supplemental Material

sj-docx-1-dhj-10.1177_20552076231194939 - Supplemental material for Optimizing the predictive power of depression screenings using machine learning Click here for additional data file. Supplemental material, sj-docx-1-dhj-10.1177_20552076231194939 for Optimizing the predictive power of depression screenings using machine learning by Yannik Terhorst, Lasse B Sander, David D Ebert and Harald Baumeister in DIGITAL HEALTH
