# Supplementary material for: Optimizing the predictive power of depression screenings using machine learning
Source: Digit Health. 2023 Aug 29;9:20552076231194939. doi: 10.1177/20552076231194939 (PMC10467308; doi:10.1177/20552076231194939)
Supplement: sj-docx-1-dhj-10.1177_20552076231194939 - Supplemental material for Optimizing the predictive power of depression screenings using machine learning [file sj-docx-1-dhj-10.1177_20552076231194939.docx]

## **Supplement 1**

## **STARD Checklist**

|  | **Section & Topic** | **No** | **Item** | **Reported on page #** |
| --- | --- | --- | --- | --- |
|  | TITLE OR ABSTRACT |  |  |  |
|  |  | 1 | Identification as a study of diagnostic accuracy using at least one measure of accuracy  (such as sensitivity, specificity, predictive values, or AUC) | 2 |
|  | ABSTRACT |  |  |  |
|  |  | 2 | Structured summary of study design, methods, results, and conclusions  (for specific guidance, see STARD for Abstracts) | 2 |
|  | INTRODUCTION |  |  |  |
|  |  | 3 | Scientific and clinical background, including the intended use and clinical role of the index test | p. 3 |
|  |  | 4 | Study objectives and hypotheses | p.4 |
|  | METHODS |  |  |  |
|  | *Study design* | 5 | Whether data collection was planned before the index test and reference standard  were performed (prospective study) or after (retrospective study) | p. 4 |
|  | *Participants* | 6 | Eligibility criteria | p. 4 |
|  |  | 7 | On what basis potentially eligible participants were identified  (such as symptoms, results from previous tests, inclusion in registry) | p. 4 |
|  |  | 8 | Where and when potentially eligible participants were identified (setting, location and dates) | p. 4 |
|  |  | 9 | Whether participants formed a consecutive, random or convenience series | p. 2 |
|  | *Test methods* | 10a | Index test, in sufficient detail to allow replication | pp. 4-7 |
|  |  | 10b | Reference standard, in sufficient detail to allow replication | pp. 4-7 |
|  |  | 11 | Rationale for choosing the reference standard (if alternatives exist) | pp. 4-7 |
|  |  | 12a | Definition of and rationale for test positivity cut-offs or result categories  of the index test, distinguishing pre-specified from exploratory | pp. 4-7 |
|  |  | 12b | Definition of and rationale for test positivity cut-offs or result categories  of the reference standard, distinguishing pre-specified from exploratory | p.5 |
|  |  | 13a | Whether clinical information and reference standard results were available  to the performers/readers of the index test | p.5 |
|  |  | 13b | Whether clinical information and index test results were available  to the assessors of the reference standard | pp.4-7 |
|  | *Analysis* | 14 | Methods for estimating or comparing measures of diagnostic accuracy | pp. 5-7 |
|  |  | 15 | How indeterminate index test or reference standard results were handled | pp. 5-7 |
|  |  | 16 | How missing data on the index test and reference standard were handled | p.5 |
|  |  | 17 | Any analyses of variability in diagnostic accuracy, distinguishing pre-specified from exploratory | pp. 5-7 |
|  |  | 18 | Intended sample size and how it was determined | p. 6 |
|  | RESULTS |  |  |  |
|  | *Participants* | 19 | Flow of participants, using a diagram | Fig. 1, p.8 |
|  |  | 20 | Baseline demographic and clinical characteristics of participants | p. 7-8 |
|  |  | 21a | Distribution of severity of disease in those with the target condition | p. 7-8 |
|  |  | 21b | Distribution of alternative diagnoses in those without the target condition | p. 7-8 |
|  |  | 22 | Time interval and any clinical interventions between index test and reference standard | n.a. |
|  | *Test results* | 23 | Cross tabulation of the index test results (or their distribution)  by the results of the reference standard | pp. 8-10 |
|  |  | 24 | Estimates of diagnostic accuracy and their precision (such as 95% confidence intervals) | pp. 8-10 |
|  |  | 25 | Any adverse events from performing the index test or the reference standard | n.a. |
|  | DISCUSSION |  |  |  |
|  |  | 26 | Study limitations, including sources of potential bias, statistical uncertainty, and generalisability | pp. 11-13 |
|  |  | 27 | Implications for practice, including the intended use and clinical role of the index test | pp. 11-13 |
|  | OTHER INFORMATION |  |  |  |
|  |  | 28 | Registration number and name of registry | p. 5 |
|  |  | 29 | Where the full study protocol can be accessed | p. 5 |
|  |  | 30 | Sources of funding and other support; role of funders | p. 13 |

Cohen, J. F., Korevaar, D. A., Altman, D. G., Bruns, D. E., Gatsonis, C. A., Hooft, L., Irwig, L., Levine, D., Reitsma, J. B., de Vet, H. C. W., & Bossuyt, P. M. M. (2016). STARD 2015 guidelines for reporting diagnostic accuracy studies: explanation and elaboration. *BMJ Open*, *6*(11). https://doi.org/10.1136/bmjopen-2016-012799

## **TRIPOD Checklist**

| **Section/Topic** | **Item** |  | **Checklist Item** | **Page** |
| --- | --- | --- | --- | --- |
| Title and abstract | | | | |
| Title | 1 | D;V | Identify the study as developing and/or validating a multivariable prediction model, the target population, and the outcome to be predicted. | P 1 |
| Abstract | 2 | D;V | Provide a summary of objectives, study design, setting, participants, sample size, predictors, outcome, statistical analysis, results, and conclusions. | P 2 |
| Introduction | | | | |
| Background and objectives | 3a | D;V | Explain the medical context (including whether diagnostic or prognostic) and rationale for developing or validating the multivariable prediction model, including references to existing models. | P 3-4 |
|  | 3b | D;V | Specify the objectives, including whether the study describes the development or validation of the model or both. | P 3-4 |
| Methods | | | | |
| Source of data | 4a | D;V | Describe the study design or source of data (e.g., randomized trial, cohort, or registry data), separately for the development and validation data sets, if applicable. | P 4 |
|  | 4b | D;V | Specify the key study dates, including start of accrual; end of accrual; and, if applicable, end of follow-up. | P 4 |
| Participants | 5a | D;V | Specify key elements of the study setting (e.g., primary care, secondary care, general population) including number and location of centres. | P 4 |
|  | 5b | D;V | Describe eligibility criteria for participants. | P 4 |
|  | 5c | D;V | Give details of treatments received, if relevant. | P 4 |
| Outcome | 6a | D;V | Clearly define the outcome that is predicted by the prediction model, including how and when assessed. | PP 5-6 |
|  | 6b | D;V | Report any actions to blind assessment of the outcome to be predicted. | P 5 |
| Predictors | 7a | D;V | Clearly define all predictors used in developing or validating the multivariable prediction model, including how and when they were measured. | P 5-7 |
|  | 7b | D;V | Report any actions to blind assessment of predictors for the outcome and other predictors. | P 5 |
| Sample size | 8 | D;V | Explain how the study size was arrived at. | P 4 |
| Missing data | 9 | D;V | Describe how missing data were handled (e.g., complete-case analysis, single imputation, multiple imputation) with details of any imputation method. | P 4 |
| Statistical analysis methods | 10a | D | Describe how predictors were handled in the analyses. | PP 5-7 |
|  | 10b | D | Specify type of model, all model-building procedures (including any predictor selection), and method for internal validation. | PP 5-7 |
|  | 10c | V | For validation, describe how the predictions were calculated. | PP 5-7 |
|  | 10d | D;V | Specify all measures used to assess model performance and, if relevant, to compare multiple models. | PP 5-7 |
|  | 10e | V | Describe any model updating (e.g., recalibration) arising from the validation, if done. | PP 5-7 |
| Risk groups | 11 | D;V | Provide details on how risk groups were created, if done. | NA |
| Development vs. validation | 12 | V | For validation, identify any differences from the development data in setting, eligibility criteria, outcome, and predictors. | PP 5-7 |
| Results | | | | |
| Participants | 13a | D;V | Describe the flow of participants through the study, including the number of participants with and without the outcome and, if applicable, a summary of the follow-up time. A diagram may be helpful. | P 7 |
|  | 13b | D;V | Describe the characteristics of the participants (basic demographics, clinical features, available predictors), including the number of participants with missing data for predictors and outcome. | P 7 |
|  | 13c | V | For validation, show a comparison with the development data of the distribution of important variables (demographics, predictors and outcome). | PP 7 |
| Model development | 14a | D | Specify the number of participants and outcome events in each analysis. | P 7-8 |
|  | 14b | D | If done, report the unadjusted association between each candidate predictor and outcome. | NA |
| Model specification | 15a | D | Present the full prediction model to allow predictions for individuals (i.e., all regression coefficients, and model intercept or baseline survival at a given time point). | PP 7-8 |
|  | 15b | D | Explain how to the use the prediction model. | P 8-9 |
| Model performance | 16 | D;V | Report performance measures (with CIs) for the prediction model. | PP 8-10 |
| Model-updating | 17 | V | If done, report the results from any model updating (i.e., model specification, model performance). | Supplement 3 |
| Discussion | | | | |
| Limitations | 18 | D;V | Discuss any limitations of the study (such as nonrepresentative sample, few events per predictor, missing data). | PP 11-13 |
| Interpretation | 19a | V | For validation, discuss the results with reference to performance in the development data, and any other validation data. | P 11-13 |
|  | 19b | D;V | Give an overall interpretation of the results, considering objectives, limitations, results from similar studies, and other relevant evidence. | PP 11-13 |
| Implications | 20 | D;V | Discuss the potential clinical use of the model and implications for future research. | PP 11-13 |
| Other information | | | | |
| Supplementary information | 21 | D;V | Provide information about the availability of supplementary resources, such as study protocol, Web calculator, and data sets. | PP 4, 11 |
| Funding | 22 | D;V | Give the source of funding and the role of the funders for the present study. | P 13 |

Collins, G. S., Reitsma, J. B., Altman, D. G., & Moons, K. G. M. (2015). Transparent reporting of a multivariable prediction model for individual prognosis or diagnosis (TRIPOD): the TRIPOD Statement. *BMC Medicine*, *13*(1). https://doi.org/10.1186/S12916-014-0241-Z

# **Supplement 2: Class imbalance and MDE status proportions in test and training set**

| **Questionnaire** | **Training** | **Testing** |
| --- | --- | --- |
| QIDS-16 | TRUE: 25.16 %  FALSE: 74.84 % | TRUE: 25.20 %  FALSE: 74.80 % |
| HAM-D-17 | TRUE: 25.20 %  FALSE: 74.80 % | TRUE: 25.20 %  FALSE: 74.80 % |
| PHQ-9 | TRUE: 24.89 %  FALSE: 75.11 % | TRUE: 25.11 %  FALSE: 74.89 % |

# **Supplement 3: Hyperparameters and Training Results**

In the training of the ML models, a logistic regression model with the QIDS-16 item scores without additional features and without dimensionality reduction or imbalanced data handling achieved a ROC AUC=0.9035490 (SE=0.005618863). Best hyperparameters were penalty=0.02702076, and mixture=0.553659. The optimal prediction threshold was 0.28 achieving a j-index of 0.66. Only the logistic regression model with the inclusion of age, gender, and the extended feature set achieved a higher AUC=0.9049435 (SE=0.005484692; hyperparameters: penalty=0.02702076, mixture=0.553659; optimal threshold: 0.302, j-index=0.66). Given the parsimoniousness of the basic item set compared to the extended model and the marginal difference in ROC AUC, we favor the basic model over the extended model.

Please see the ROC AUC result plot of all 261 candidate models below. High resolution version of the training results plot is accessible at: <https://osf.io/3hnvz/>.


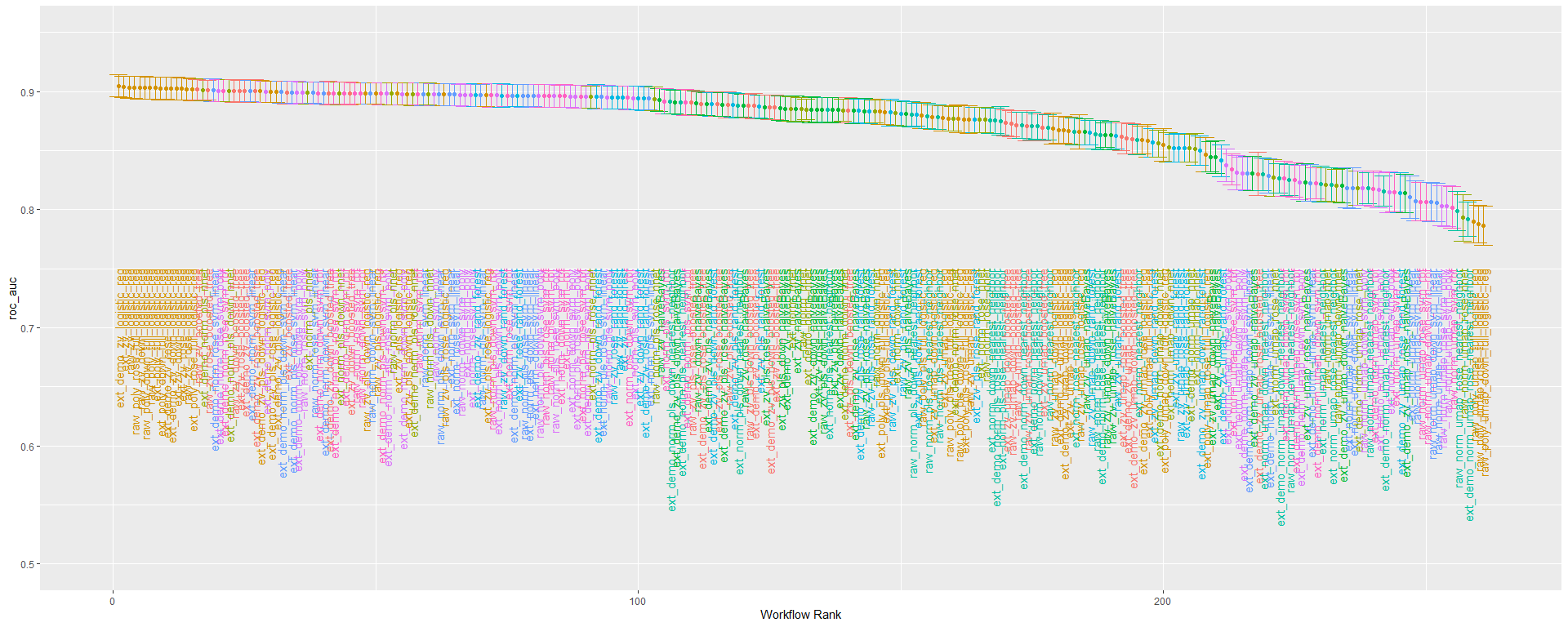


In the training of the ML models, a random forest model with the HAM-D-17 item scores without additional features and without dimensionality reduction or imbalanced data handling achieved the best performance ROC AUC= 0.8776405 (SE=0.005836733). The best hyperparameters were mtry=2, trees=1481, and min_n=23. The optimal prediction threshold was 0.32 achieving a j-index of 0.60.

Please see the ROC AUC result plot of all 261 candidate models below. High resolution version of the training results plot is accessible at: <https://osf.io/3hnvz/>.


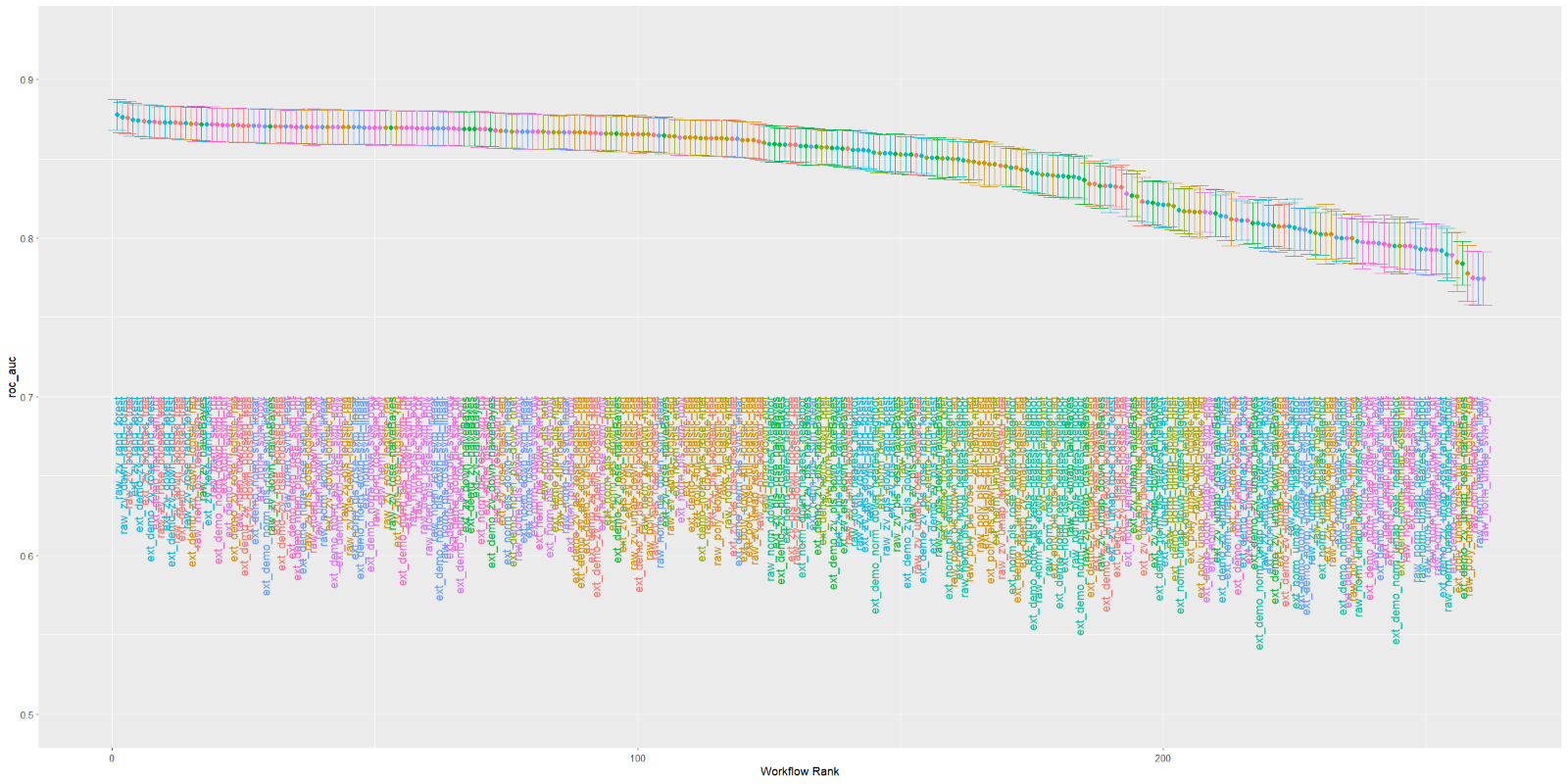


In the training of the ML models, a naïve Bayes model PHQ-9 item scores and the extended feature set, but without dimensionality reduction or imbalanced data handling achieved the best performance ROC AUC=0.8446753 (SE=0.007522153). The best hyperparameters were smoothness=0.7130864, and Laplace=2.700569. The optimal prediction threshold was 0.006 achieving a j-index of 0.60.

Please see the ROC AUC result plot of all 261 candidate models below. High resolution version of the training results plot is accessible at: <https://osf.io/3hnvz/>.


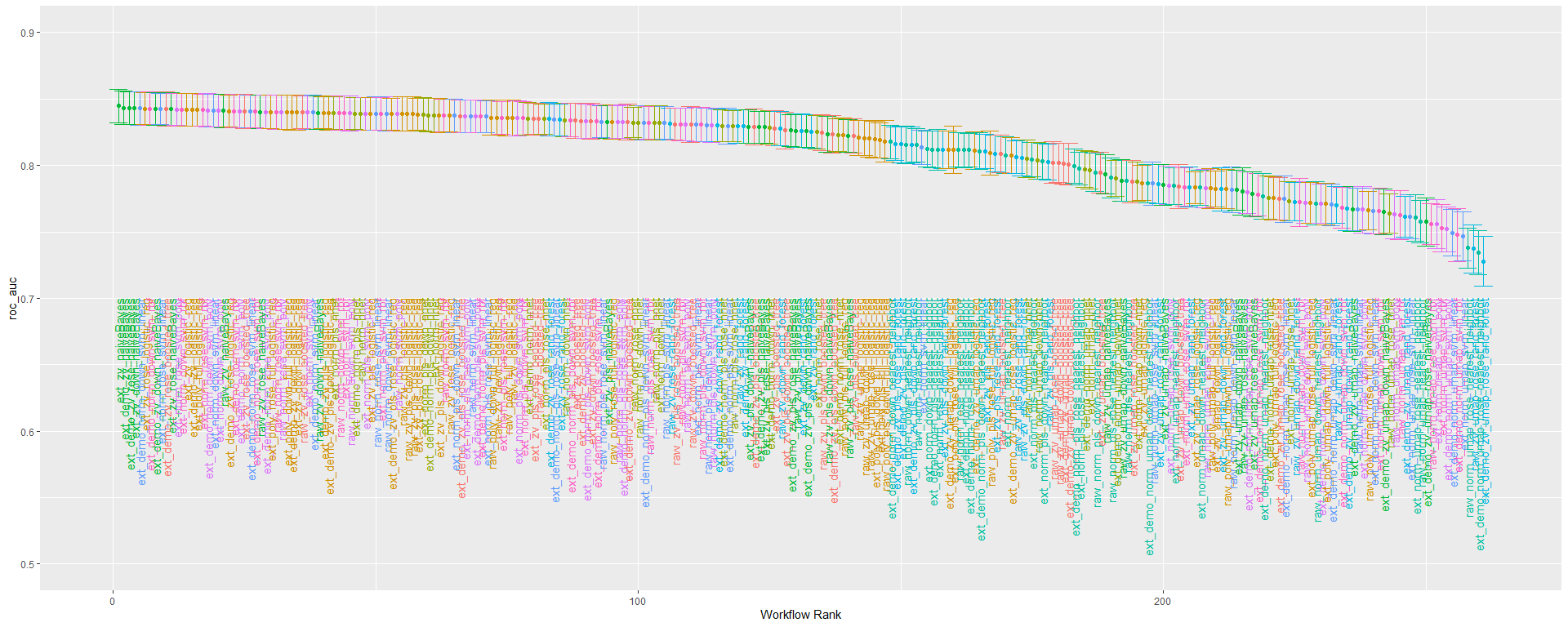


# **Supplement 4: Tuning overview**

- bag_tree(cost_complexity = tune(), tree_depth = tune(), min_n = tune(),class_cost = tune())
- bag_mars(num_terms = tune(), prod_degree = tune(),prune_method = tune())
- bart(trees = tune(), prior_terminal_node_coef = tune(), prior_terminal_node_expo = tune(), prior_outcome_range = 2)
- boost_tree(tree_depth = tune(), learn_rate = tune(), loss_reduction = tune(), min_n = tune(), sample_size = tune(), trees = tune(), mtry = tune()); mtry was set between 1 to 20
- decision_tree(cost_complexity = tune(), min_n = tune(), tree_depth = tune()); mtry was set between 1 to 20
- logistic_reg(penalty = tune(), mixture = tune())
- mars(prod_degree = tune(),num_terms = tune(), prune_method = tune())
- mlp(hidden_units = tune(), penalty = tune(), epochs = tune()); hidden unit was set between 1 to 27
- naïve_Bayes(smoothness = tune(),Laplace = tune())
- nearest_neighbor(neighbors = tune(), dist_power = tune(), weight_func = tune())
- rand_forest(mtry = tune(), min_n = tune(), trees = tune()); mtry was set between 1 to 20
- svm_linear(cost = tune(), margin = tune())
- svm_poly(cost = tune(), degree = tune(), scale_factor = tune(), margin = tune())
- svm_rbf_spec <- svm_rbf(cost = tune(), rbf_sigma = tune(), margin = tune())

Full code for model specifications, recipes for preprocessing, and workflow sets can be accessed as the open science framework <https://osf.io/3hnvz/>.

Models were fitted using the parsnip package using the tidymodels framework. Please see the code at OSF for the used engines respectively. Additional, information on the tuning parameters can be found at: <https://www.tidymodels.org/find/parsnip/>

# **Supplement 5: Session Information**

R version 4.2.2 (2022-10-31 ucrt)

Platform: x86_64-w64-mingw32/x64 (64-bit)

Running under: Windows 10 x64 (build 22621)

Matrix products: default

locale:

[1] LC_COLLATE=German_Germany.utf8 LC_CTYPE=German_Germany.utf8

[3] LC_MONETARY=German_Germany.utf8 LC_NUMERIC=C

[5] LC_TIME=German_Germany.utf8

attached base packages:

[1] parallel stats graphics grDevices utils datasets

[7] methods base

other attached packages:

[1] ggforce_0.4.1 corrplot_0.92

[3] gridExtra_2.3 ROCit_2.1.1

[5] cutpointr_1.1.2 doParallel_1.0.17

[7] doSNOW_1.0.20 snow_0.4-4

[9] iterators_1.0.14 foreach_1.5.2

[11] dbarts_0.9-23 klaR_1.7-2

[13] kknn_1.3.1 kernlab_0.9-32

[15] nnet_7.3-18 xgboost_1.7.5.1

[17] glmnet_4.1-7 Matrix_1.5-4

[19] earth_5.3.2 plotmo_3.6.2

[21] TeachingDemos_2.12 plotrix_3.8-2

[23] Formula_1.2-5 ranger_0.15.1

[25] embed_1.1.0 fastICA_1.2-3

[27] mixOmics_6.23.4 lattice_0.20-45

[29] MASS_7.3-60 learntidymodels_0.0.0.9001

[31] lubridate_1.9.2 forcats_1.0.0

[33] tidyverse_2.0.0 probably_0.1.0

[35] vip_0.3.2 finetune_1.1.0

[37] themis_1.0.1 discrim_1.0.1

[39] baguette_1.0.1 yardstick_1.2.0

[41] workflowsets_1.0.1 workflows_1.1.3

[43] tune_1.1.1 tidyr_1.3.0

[45] tibble_3.2.1 rsample_1.1.1

[47] recipes_1.0.6 purrr_1.0.1

[49] parsnip_1.1.0 modeldata_1.1.0

[51] infer_1.0.4 ggplot2_3.4.2

[53] dials_1.2.0 scales_1.2.1

[55] broom_1.0.4 tidymodels_1.1.0

[57] stringr_1.5.0 stringi_1.7.12

[59] dplyr_1.1.2 readxl_1.4.2

[61] readr_2.1.4

loaded via a namespace (and not attached):

[1] utf8_1.2.3 questionr_0.7.8 reticulate_1.28

[4] tidyselect_1.2.0 grid_4.2.2 combinat_0.0-8

[7] BiocParallel_1.32.6 pROC_1.18.2 munsell_0.5.0

[10] codetools_0.2-18 future_1.32.0 miniUI_0.1.1.1

[13] withr_2.5.0 keras_2.11.1 colorspace_2.1-0

[16] highr_0.10 knitr_1.42 rstudioapi_0.14

[19] tensorflow_2.11.0 listenv_0.9.0 labeling_0.4.2

[22] polyclip_1.10-4 farver_2.1.1 DiceDesign_1.9

[25] parallelly_1.35.0 vctrs_0.6.2 generics_0.1.3

[28] ipred_0.9-14 xfun_0.39 timechange_0.2.0

[31] R6_2.5.1 lhs_1.1.6 cachem_1.0.8

[34] promises_1.2.0.1 gtable_0.3.3 Cubist_0.4.2.1

[37] globals_0.16.2 timeDate_4022.108 rlang_1.1.1

[40] zeallot_0.1.0 splines_4.2.2 yaml_2.3.7

[43] reshape2_1.4.4 backports_1.4.1 httpuv_1.6.11

[46] inum_1.0-5 tools_4.2.2 lava_1.7.2.1

[49] ellipsis_0.3.2 RColorBrewer_1.1-3 proxy_0.4-27

[52] Rcpp_1.0.10 plyr_1.8.8 base64enc_0.1-3

[55] rpart_4.1.19 haven_2.5.2 ggrepel_0.9.3

[58] furrr_0.3.1 magrittr_2.0.3 data.table_1.14.8

[61] RSpectra_0.16-1 GPfit_1.0-8 mvtnorm_1.1-3

[64] whisker_0.4.1 ROSE_0.0-4 matrixStats_0.63.0

[67] hms_1.1.3 mime_0.12 evaluate_0.21

[70] xtable_1.8-4 shape_1.4.6 tfruns_1.5.1

[73] compiler_4.2.2 ellipse_0.4.5 htmltools_0.5.5

[76] corpcor_1.6.10 later_1.3.1 tzdb_0.4.0

[79] libcoin_1.0-9 tweenr_2.0.2 cli_3.6.1

[82] C50_0.1.8 gower_1.0.1 igraph_1.4.2

[85] pkgconfig_2.0.3 rARPACK_0.11-0 hardhat_1.3.0

[88] prodlim_2023.03.31 digest_0.6.31 rmarkdown_2.21

[91] cellranger_1.1.0 uwot_0.1.14 shiny_1.7.4

[94] lifecycle_1.0.3 jsonlite_1.8.4 viridisLite_0.4.2

[97] fansi_1.0.4 labelled_2.11.0 pillar_1.9.0

[100] fastmap_1.1.1 survival_3.4-0 glue_1.6.2

[103] conflicted_1.2.0 png_0.1-8 class_7.3-20

[106] partykit_1.2-20 memoise_2.0.1 e1071_1.7-13

[109] future.apply_1.10.0

**Machine details:**

Processor AMD Ryzen 7 5800X 8-Core Processor, 3801 Mhz, 8 Core(s), 16 Logical Processor(s)

Installed Physical Memory (RAM) 16,0 GB

Total Physical Memory 15,9 GB

Total Virtual Memory 24,5 GB

# **Supplement 5: Feature Importance**

**QIDS-16**

Variable importance plot for the QIDS-16 classifier limited to the original QIDS-16 items.

Absolute regression coefficients


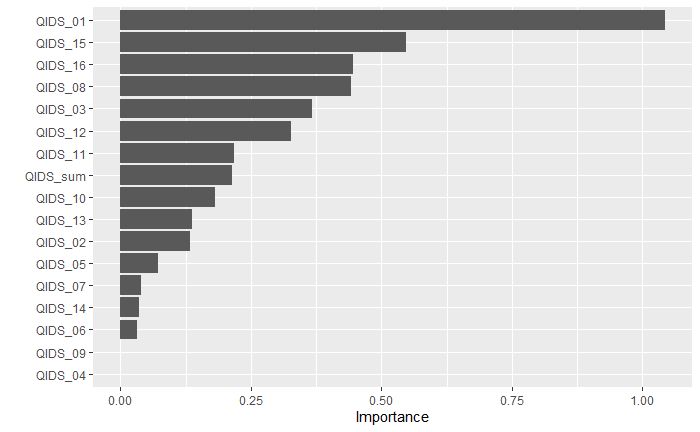


Variable importance plot for the QIDS-16 classifier with the extended feature set, age, and gender.

Absolute regression coefficients


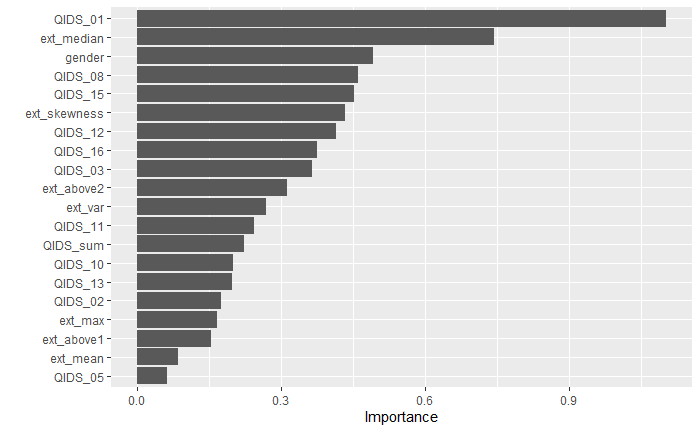


**HAM-D-17**

Variable importance plot for the HAM-D-17 classifier.

Gini impurity index


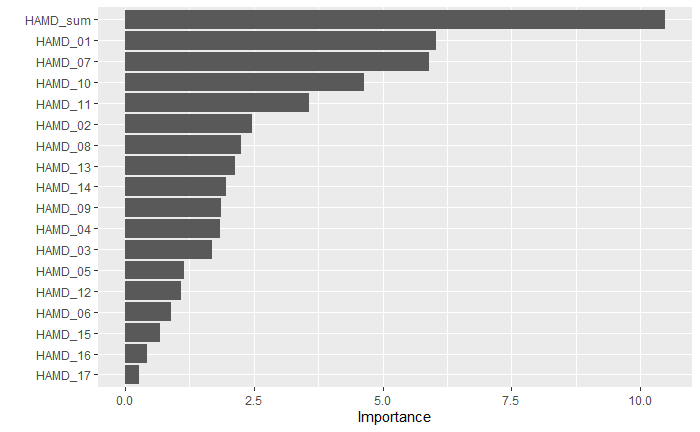


**PHQ-9**

The used analysis package does not provide a visual feature importance plot. Hence, feature importance can only be inferred from the candidate models (see supplement 3). The extended feature set outperformed the raw PHQ-9 item feature set indicating the relevance of the engineered features. In contrast, the addition of demographic variables (i.e., age, gender) did harm the performance showing low importance of age and gender.

# **Supplement 6: Confusion matrix**

All confusion matrices refer to the performance in the testing set.

**QIDS-16 – basic (logistic regression)**

Truth

Prediction 1 0

1 55 23

0 9 167

**QIDS-16 – extended, age, and gender (logistic regression)**

Truth

Prediction 1 0

1 53 21

0 11 169

**HAM-D-17 – best ML (random forest)**

Truth

Prediction 1 0

1 48 26

0 16 164

**PHQ-9 – best ML (naïve Bayes)**

Truth

Prediction 1 0

1 45 46

0 12 124
